# Structural Characterization and In Vivo Anti-Inflammatory Activity of Fucoidan from *Cystoseira crinita* (Desf.) Borry

**DOI:** 10.3390/md20110714

**Published:** 2022-11-15

**Authors:** Elisaveta Apostolova, Paolina Lukova, Alexandra Baldzhieva, Cédric Delattre, Roland Molinié, Emmanuel Petit, Redouan Elboutachfaiti, Mariana Nikolova, Ilia Iliev, Marianna Murdjeva, Vesela Kokova

**Affiliations:** 1 Department of Pharmacology, Toxicology, and Pharmacotherapy, Faculty of Pharmacy, Medical University-Plovdiv, Vasil Aprilov Str. 15A, 4002 Plovdiv, Bulgaria; 2 Department of Pharmacognosy and Pharmaceutical Chemistry, Faculty of Pharmacy, Medical University-Plovdiv, Vasil Aprilov Str. 15A, 4002 Plovdiv, Bulgaria; 3 Department of Microbiology and Immunology, Faculty of Pharmacy, Medical University-Plovdiv, Vasil Aprilov Str. 15A, 4002 Plovdiv, Bulgaria; 4 Research Institute at Medical University-Plovdiv, Vasil Aprilov Str. 15A, 4002 Plovdiv, Bulgaria; 5 Clermont Auvergne INP, CNRS, Institut Pascal, Université Clermont Auvergne, 63000 Clermont-Ferrand, France; 6 Institut Universitaire de France (IUF), 1 rue Descartes, 75005 Paris, France; 7 UMRT INRAE 1158 BioEcoAgro, BIOlogie des Plantes et Innovation (BIOPI), Avenue des Facultés, IUT d’Amiens, Université de Picardie Jules Verne, Le Bailly, 80025 Amiens, France; 8 Department of Biochemistry and Microbiology, Faculty of Biology, Plovdiv University Paisii Hilendarski, Tsar Asen Str. 24, 4000 Plovdiv, Bulgaria

**Keywords:** fucoidan, *Cystoseira crinita*, TNF-α, IL-1β, peritonitis, rat paw edema, cytokines, anti-inflammatory effect

## Abstract

The aim of this study was to evaluate the effects of fucoidan isolated from *C. crinita* on histamine-induced paw inflammation in rats, and on the serum levels of TNF-α, IL-1β, IL-6, and IL-10 in rats during systemic inflammation response. The levels of TNF-α in a model of acute peritonitis in rats were also investigated. The isolated crude fucoidan was identified as a sulfated xylogalactofucan with high, medium, and low molecular weight fractions and a content of fucose of 39.74%, xylose of 20.75%, and galactose of 15.51%. Fucoidan from *C. crinita* showed better anti-inflammatory effects in the rat paw edema model, and this effect was present during all stages of the experiment. When compared to controls, a commercial fucoidan from *F. vesiculosus,* the results also displayed anti-inflammatory activity on the 60th, 90th, and 120th minute of the experiment. A significant decrease in serum levels of IL-1β in rats treated with both doses of *C. crinita* fucoidan was observed in comparison to controls, whereas TNF-α concentrations were reduced only in the group treated with fucoidan from *C. crinita* at the dose of 25 mg/kg bw. In the model of carrageenan-induced peritonitis, we observed a tendency of decrease in the levels of the pro-inflammatory cytokine TNF-α in peritoneal fluid after a single dose of *C. crinita* fucoidan, but this did not reach the statistical significance margin. Single doses of *C. crinita* fucoidan did not alter serum levels of the anti-inflammatory cytokine IL-10 in animals with lipopolysaccharide-induced systemic inflammation.

## 1. Introduction

The inflammation, as an initial response of the immune system, could occur under the influence of harmful stimuli such as injury, stress, or infections. These stimuli (e.g., bacterial endotoxin lipopolysaccharides (LPS) and other foreign antigens) cause the migration of macrophages and neutrophils to the site of contact. The activated cells produce and release pro-inflammatory mediators such as tumor necrosis factor-α (TNF-α), interleukin-1β (IL-1β), and interleukin 6 (IL-6). The increased levels of these substances promote prolonged inflammation [1,2]. These cytokines induce more infiltration of monocytes, granulocytes, lymphocytes, and mast cells at the site of injury, which aims at antigen elimination and tissue restoration. Augmented infiltration and activation of these cells is related to an increased risk of tissue damage due to excessive inflammation and its main symptoms, such as pain and edema [3,4].

*Cystoseira crinita* (*C. crinita*) is a brown macroalgae with wide distribution in the Mediterranean region and the Black Sea. Even though some research on *C. crinita* from Mediterranean coasts has been performed, the brown macroalgae from the Black Sea region has been left out of scope. Moreover, the pharmacological properties of fucoidan, derived from this species, remain unknown.

Fucoidans are a group of sulfated polysaccharides, often detected in the cell walls of brown seaweed and other marine species [5]. Recently, fucoidans derived from algae have been the subject of much research regarding their multiple biological activities and possible therapeutic potential. Several research studies focus on their various pharmacological effects, including antitumor, immunomodulatory, anti-viral, anti-microbial, anti-diabetes, nephroprotective, anti-oxidant, anti-inflammatory, and anti-coagulant effects [1,6,7,8].

However, the development of standardized fucoidan supplements is a complicated process due to their complex chemical composition. The chemical composition is greatly influenced by the source, species, geographic location, and extraction process. The activity of the sulfated polysaccharides depends not only on the composition but also on the molecular weight, the structure of the molecules, and the route of administration. Moreover, fucoidans, isolated from the same algal source, could have the opposite effect when tested on different animal models.

The aim of this study is to identify the chemical composition and structure of fucoidan isolated from *C. crinita* and to evaluate its effects on an experimental model of paw inflammation in rats, and on the serum levels of TNF-α, IL-1β, IL-6, and interleukin 10 (IL-10) in rats with systemic inflammation. The levels of TNF-α in a model of acute peritonitis in rats were also investigated.

## 2. Results

### 2.1. Extraction Yield and Chemical Composition

The chemical content and extraction yield of *C. crinita* fucoidan are presented in Table 1. The extraction yield of *C. crinita* fucoidan was 5.15%, calculated as a percent of the dry weight of the alga. Colorimetric assays revealed that the tested fucoidan contained mainly neutral sugars (46.64%) and a minor amount of uronic acids (13.15%). The sulfate content was relatively low (17%), a characteristic of fucoidan for the *Cystoseira* genus compared to other brown algae genus [9,10]. Polyphenolic (<0.10%) and protein content (0.56%) were low due to the pre-extraction and purification steps of the dried, pulverized algal material.

The monosaccharide composition of *C. crinita* crude fucoidan and a standard commercial sample from *F. vesiculosus* (Sigma-Aldrich, Saint Louis, MO, USA) were analyzed by HPAEC-PAD after chemical hydrolysis using trifluoroacetic acid (TFA). The monosaccharide contents were expressed in terms of a molar percentage of the total monosaccharides detected.

As mentioned in Table 2, crude fucoidan extracted from *C. crinita* was principally composed of fucose (39.74%), xylose (20.75%), galactose (15.51%), and glucuronic acid (13.52%), but also contained small amounts of glucose (5.50%), rhamnose (2.37%), and arabinose (2.13%). The sugar profiles of uronic acid analysis have noted the presence of two other monosaccharide residues, mannuronic and guluronic acids, in *C. crinita* crude fucoidan and fucoidan standard with retention times of 18.25 and 19.10 min, respectively. The relative retention times of these two uronic acids were different from those obtained with glucuronic and galacturonic acids being analyzed under the same conditions [9,11,12]. Without access to mannuronic and guluronic acid standards, it was not possible to accurately quantify these two minor constituents.

A structural comparison with a standard fucoidan from *F. vesiculosus* (Sigma-Aldrich), carried out under the same experimental analysis conditions as described above, confirmed that fucose was the most represented sugar among the neutral monosaccharides forming the structure of the standard fucoidan and the crude fucoidan isolated from *C. crinita* (Table 2). In addition, the amount of fucose (55.69%) was higher in the standard fucoidan sample than in the crude fucoidan extract (39.74%). The decrease in the amount of fucose in *C. crinita* crude fucoidan was compensated by an increase in other sugars such as rhamnose, arabinose, galactose, and glucose.

### 2.2. FTIR Spectroscopy Analysis

**Figure 1:**
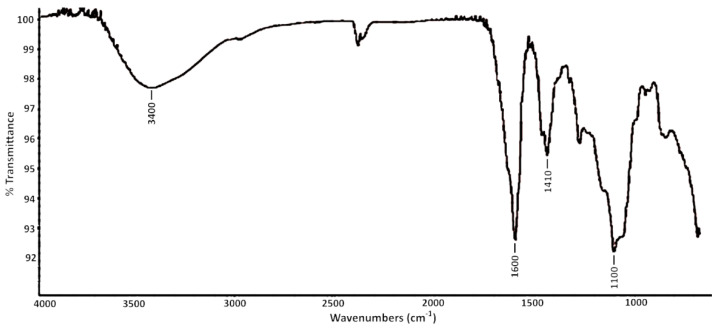
FTIR of fucoidan extracted from *C. crinita*.

The Fourier-transform infrared (FTIR) spectrum of *C. crinita* crude fucoidan is shown in Figure 1. The band at 3427 cm^−1^ was associated to O-H stretching of sugar residues [13]. The absorption signal at 1611 cm^−1^ was attributed to the vibration of (C=O) ester groups in the acid residues, which confirmed the presence of uronic acids [14]. The peak obtained at 1412 cm^−1^ could be assigned to the stretching of -CH_2_ groups of neutral monosaccharides and to the -CH_3_ groups of the fucosyl residues [9]. The band observed at 1135 cm^−1^ could be ascribed to the stretching models of pseudosymmetric sulfate groups (O=S=O) and the hemiacetal groups of fucosyl residues [9,15].

### 2.3. Proton Nuclear Magnetic Resonance (^1^H NMR) Spectroscopy

The structural characterization of fucoidan was assessed by ^1^H NMR analysis. From our NMR data, the signal attribution was based on the interpretation, identification, and comparison of the ^1^H NMR spectrum of native fucoidan polysaccharides obtained in previous studies, which included ours [10,16,17].

**Figure 2:**
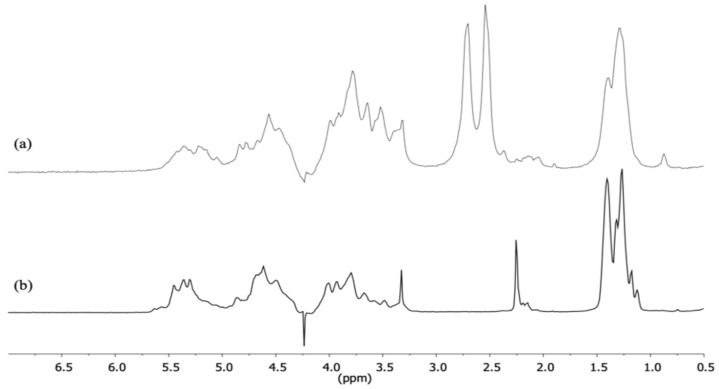
^1^H NMR spectra of the sulfated fucoidan polysaccharides (**a**) *C. crinita* crude fucoidan and (**b**) commercial standard fucoidan from *F. vesiculosus* at 80 °C in D_2_O solution.

Firstly, the acquired spectra of the fucoidans had a low resolution, essentially due to their complex sulfated heterogeneous structure. However, there were strong similarities between the ^1^H NMR spectra of the isolated fucoidan from *C. crinita* (crude fucoidan) and the standard commercial fucoidan (Figure 2).

Secondly, the two spectra exhibited five regions characteristic of fucoidans. The intense peaks at 1.2 and 1.4 ppm were assigned to the methyl group (CH_3_), which is the main characteristic of the fucopyranose unit. The residues with the signals at 1.2 ppm may be attributed to *α*-(1→3)-linked L-fucosyl residues. Methyl signals appearing around 1.40 ppm can be assigned to *α*-(1→4)-linked L-fucose [18]. The signals at 2.2 ppm observed only in the spectra of standard fucoidans referred to CH_3_ protons of *O*-acetyl groups, which are frequently detected in algal polysaccharides. These signals were absent in the spectrum of crude fucoidan isolated in this study from *C. crinita*. Strong signals detected around 2.5 and 2.7 ppm in the crude fucoidan spectrum could be correlated, on the one hand, in agreement with the literature, to the functional group acetyl amine of the hexose (*N*-acetyl-galactosamine) or pentose (*N*-acetyl-fucosamine) sugar moiety [19]. On the other hand, these strong signals could be assigned to the presence of functional groups such as amino acids, carboxylic acids, alcohols, or phenols in biomolecules (proteins, polysaccharides, polyphenols, and other compounds) present in the crude fucoidan extracts [20]. The ^1^H NMR signals, which ranged between 3.5–4.0 ppm, could be attributed to H2–H6 protons of sugar residues.

Finally, it is well known that the chemical shift of the anomeric proton signal corresponds to *α*-type when it is >5 ppm and to *β*-type when it is <5 ppm [21]. So, the signal region between 5.3 and 5.6 ppm denoted *α*-anomeric protons, which appeared as two broad unresolved multiplets centered at 5.3 and 5.4 ppm, with two additional small resolved doublets at 5.5 and 5.6 ppm. The high-field signal at 4.5 ppm may be assigned as *β*-D-galactopyranose residue. Similar chemical shift regions were reportedly observed previously in the ^1^H NMR spectra of polymers of *α*-linked L-fucopyranose and *β*-D-galacto-xylopyranose and other sugar units incorporated into the fucoidan polymers.

### 2.4. SEC-MALS Analysis

**Figure 3:**
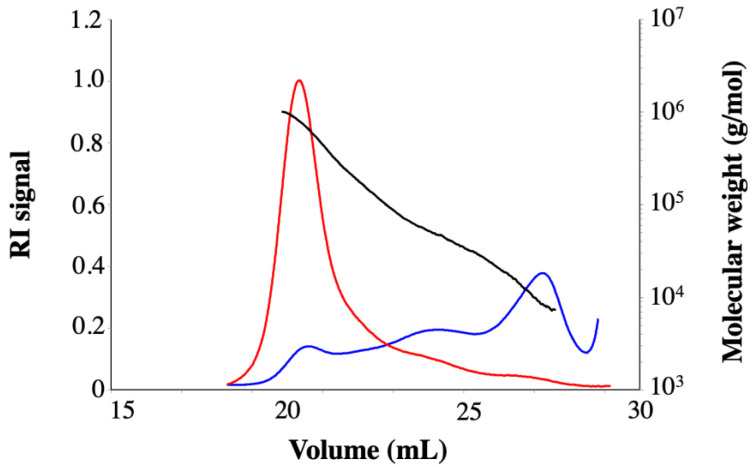
SEC-MALS chromatogram of *C. crinita* crude fucoidan giving Mw (g/mol) versus V (mL) (black), RI signal (blue), and light scattering at 90° (red).

Size-exclusion chromatography-Multi-Angle Light Scattering (SEC/MALS) experiments were carried out in 0.1 mol/l NaNO_3_ to determine the molecular weights of the sulfated fucoidans studied. Figure 3 reports the elution profiles and thus the molecular weight distributions of *C. crinita* crude fucoidan.

Light scattering (LS; 90°) and refractive index (RI) signals provide qualitative information about the solution state of the system being examined. Thus, the LS signal of fucoidan around 19 mL is correlated with the RI signal, which indicates that the solution studied does not present any aggregates. Moreover, at the end of elution (29 mL), the intensity of the LS signal returns to the initial level, indicating that there is no tailing phenomenon of the compound analyzed (no interaction with the column packing material, elution according to their hydrodynamic sizes). The polysaccharides were eluted between 19 and 28 mL, and three populations were detected: a high molecular weight fraction (Mw = 5.34 × 10^5^ g/mol), a medium molecular weight fraction (Mw = 7.01 × 10^4^ g/mol), and a low molecular weight fraction (Mw = 1.38 × 10^4^ g/mol) (Table 3). This large distribution was noted in the literature, with similar tendencies, for fucoidan extracted from the same species [22]. It was also mentioned that the molecular size of fucoidans varies between 13 and 950 kDa, depending on the origin of macroalgae [23]. Furthermore, age, geographical origin, season of harvesting, and the extraction method can influence the physicochemical characteristics and biological activity of fucoidans.

### 2.5. Effect of Fucoidan on Histamine-Induced Paw Edema in Rats

**Figure 4:**
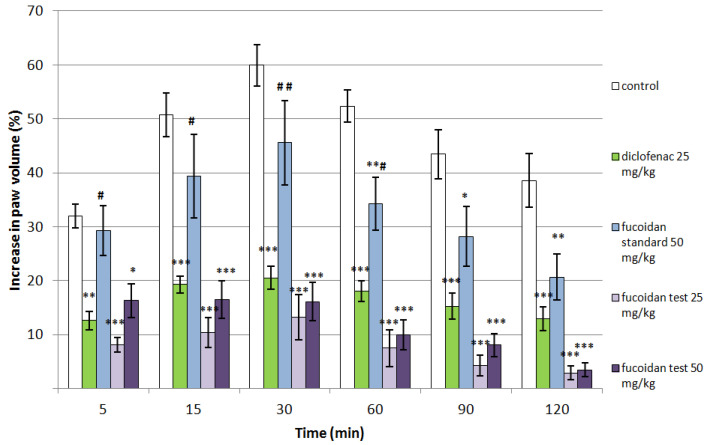
Effects of diclofenac, fucoidan standard from *F.vesiculosus* (50 mg/kg bw), and fucoidan test from *C. crinita* (25 and 50 mg/kg bw) on paw edema induced by histamine in rats. * *p* < 0.05 vs. controls at the same time; ** *p* < 0.01 vs. controls at the same time; *** *p* < 0.001 vs. controls at the same time; ^#^ *p* < 0.05 vs. diclofenac at the same time; ^##^ *p* < 0.01 vs. diclofenac at the same time.

Fucoidan from *C. crinita* showed well-defined anti-inflammatory effects in a model of histamine-induced paw edema in rats, and this effect was present during all stages of the experiment (Figure 4). Treatment with the lower dose of fucoidan (25 mg/kg) significantly decreased the paw edema after 5 min of the experiment in comparison to controls (8.08 ± 1.41 vs. 31.94 ± 2.23; *p* < 0.001). Similar results were observed with the higher dose (16.28 ± 3.09 vs. 31.94 ± 2.23; *p* < 0.05). A significant anti-phlogistic effect of both (25 mg/kg and 50 mg/kg) tested doses of fucoidan was also registered 15 min (10.36 ± 2.77 and 16.52 ± 3.49 vs. 50.72 ± 4.05; *p* < 0.001) and 30 min (13.20 ± 4.24 and 16.06 ± 3.56 vs. 59.94 ± 3.85; *p* < 0.001) after the histamine injection when compared to controls at the same time points. After 60 min of the experiment, the anti-inflammatory effect of both doses of *C. crinita* fucoidan was also present (7.49 ± 3.43 and 9.94 ± 2.83 vs. 52.32 ± 2.98; *p* < 0.001). These effects persisted until the end of the experiment and the values at the 90th and 120th min in comparison to controls, which were 4.21 ± 1.93 vs. 43.44 ± 4.52 (*p* < 0.001) and 2.85 ± 1.30 vs. 38.58 ± 5.03 (*p* < 0.001) for the lower dose fucoidan, and 8.06 ± 2.10 vs. 43.44 ± 4.52 (*p* < 0.001) and 3.45 ± 1.26 vs. 38.58 ± 5.03 (*p* < 0.001) for the higher dose fucoidan. When compared to controls, fucoidan from *F. vesiculosus* also showed anti-inflammatory activity at the 60th min (34.27 ± 4.90 vs. 52.32 ± 2.98; *p* < 0.01), at the 90th min (28.18 ± 5.51 vs. 43.44 ± 4.52; *p* < 0.05), and at the 120th minute of the testing (20.65 ± 4.31 vs. 38.58 ± 5.03; *p* < 0.01). The isolated fucoidan from *C. crinita* was more active in comparison to the standard (fucoidan from *F. vesiculosus*) during the late stages of the inflammation (15 to 120 min; Table 4).

### 2.6. Changes in Pro-Inflammatory Cytokine Levels (TNF-α, IL-1β and IL-6) in Serum and Peritoneal Fluid

**Figure 5:**
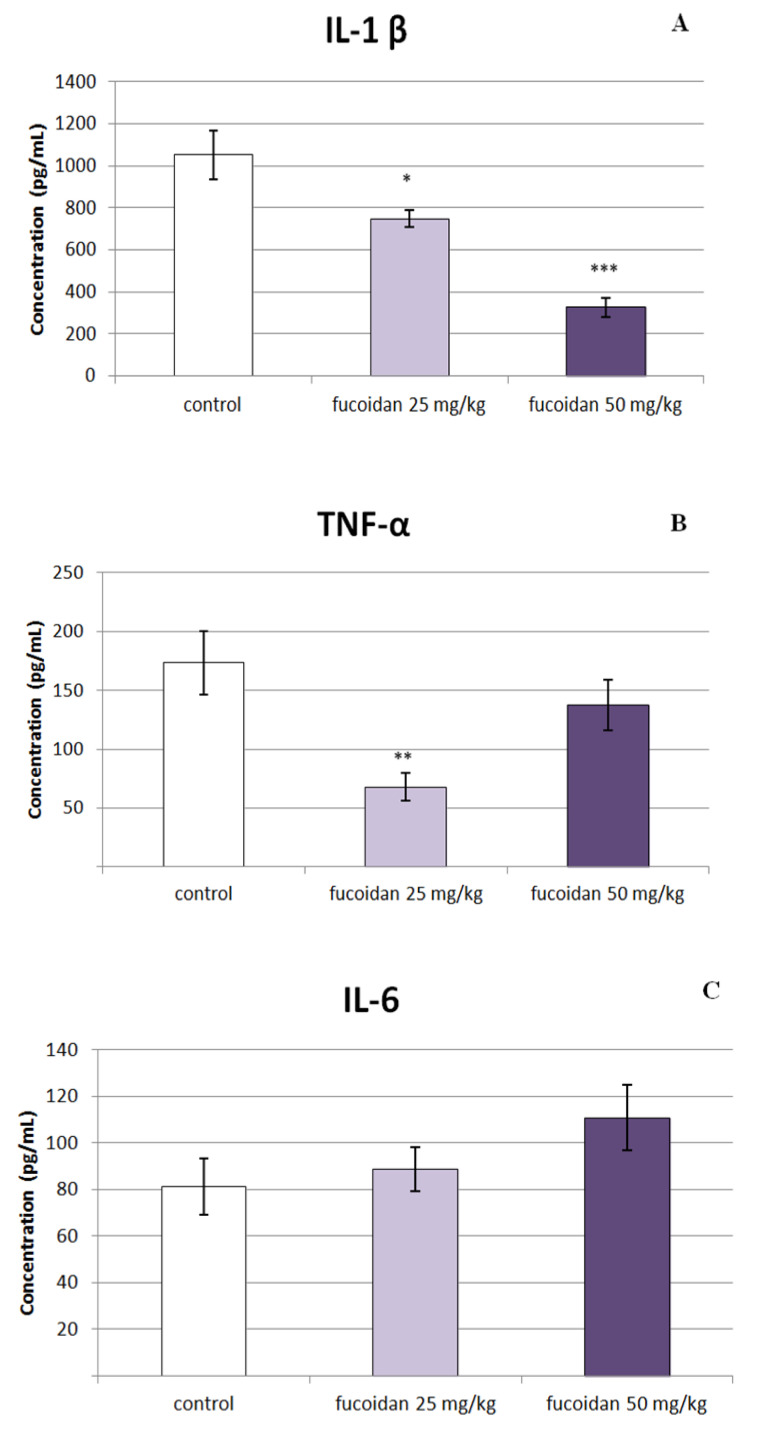
Effect of a single application of fucoidan from *C. crinita* (25 and 50 mg/kg bw) on serum levels of the pro-inflammatory cytokines IL-1β (panel **A**), TNF-α (panel **B**), and Il-6 (panel **C**) in LPS-induced systemic inflammation in rats. * *p* < 0.05 vs. same cytokine controls; ** *p* < 0.01 vs. same cytokine controls; *** *p* < 0.001 vs. same cytokine controls.

As shown in Figure 5A, a significant decrease in IL-1β levels in the serum of rats treated with both doses of *C. crinita* fucoidan in comparison to controls was observed; however, the effect was dose-dependent, and the decrease was more prominent in rats treated with the higher dose of fucoidan (50 mg/kg bw). The estimated levels were 747.67 ± 40.26 vs. 1052.58 ± 114.71 (*p* < 0.05) for the lower dose and 327.55 ± 45.61 vs. 1052.58 ± 114.71 (*p* < 0.001) for the higher dose, respectively. This decreasing effect was observed also in TNF-α serum levels of animals treated with fucoidan from *C. crinita* in a dose of 25 mg/kg bw in comparison to controls (67.86 ± 11.58 vs. 173.48 ± 26.83; *p* < 0.01; Figure 5B). No differences in the IL-6 levels were observed after a single dose of fucoidan (Figure 5C).

**Figure 6:**
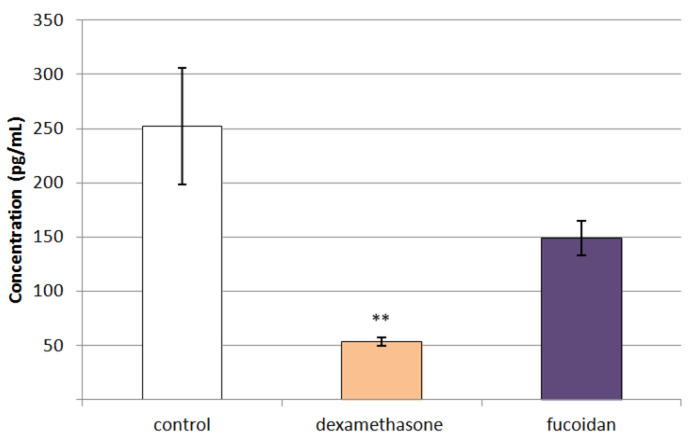
Effect of a single application of dexamethasone (0.2 mg/kg bw) and fucoidan from *C. crinita* (25 mg/kg bw) on the levels of TNF-α in the peritoneal fluid of rats with carrageenan-induced peritonitis. ** *p* < 0.01 vs. saline-treated controls.

We observed a tendency towards lower levels of the pro-inflammatory cytokine TNF-α in the peritoneal fluid of rats with a model of peritonitis after a single dose of *C. crinita* fucoidan, but these effects did not reach statistical significance (Figure 6).

### 2.7. Changes in Anti-Inflammatory Cytokine Levels (IL-10) in Serum

**Figure 7:**
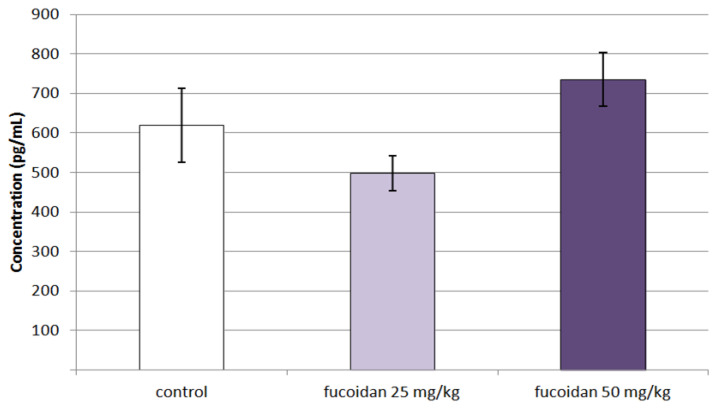
Effects of a single dose of fucoidan from *C. crinita* (25 and 50 mg/kg bw) on serum levels of IL-10 in rats with LPS-induced systemic inflammation.

Single doses of *C. crinita* fucoidan did not alter serum levels of the anti-inflammatory cytokine IL-10 in animals with lipopolysaccharide-induced systemic inflammation. This observation is supported by the data shown in Figure 7.

## 3. Discussion

Brown algae fucoidan has been the subject of intensive research. Many scientific reports proved that the biological activities of fucoidans were intimately dependent on the extraction process, chemical structure, and molecular weight [24]. Algae fucoidan is usually found to form complexes with different molecules, such as polyphenols, proteins, lipids, and other polysaccharides (alginates), tightly adsorbed to it during the process of extraction [25]. Therefore, an appropriate pre-treatment procedure is needed to achieve high purity in the final product. In the present study, a pre-treatment procedure with ethanol:formaldehyde:water (80:5:15, *v/v/v*) was chosen due to the ability of formaldehyde to link and fix phenols and make them insoluble. Furthermore, the ethanol:water solution prevents the extraction of fucoidan during the purification procedure and leads to a higher polysaccharide yield [25].

Fucoidans can be obtained by multiple-step extraction using diluted mineral acids, water, or enzymes or by some novel techniques, such as microwave- or ultrasound-assisted extraction [24]. In the current study, a dilute acid extraction of fucoidan was preferred due to the high production yield obtained (5.15%), the cost-efficiency of this method, and its potential application for industrial valorization of the abundant macroalgae *C. crinita* from the Bulgarian Black Sea coast. The obtained fucoidan had a sulfate content (17%) like previously reported *Cystoseira* sp. fucoidans extracted through different methods, for example: *C. compressa* (14.65%), *C. barbata* (22.51%) and *C. costata* (23.2%), obtained using acid extraction [9,26,27], and *C. sedoides* (16.3%), *C. crinita* (15.7%), and *C. compressa* fucoidan (16.6%), obtained using sequential extraction with an aqueous solution of CaCl_2_ [28].

Contrary to other fucoidans that typically contained only fucose as the major neutral monosaccharide, fucoidan extracts isolated from different species of the genus *Cystoseira* (*C. compressa*, *C. barbata*, and *C. costata*) have been known as sulfated galactofucans possessing high content of fucose and galactose [29]. The fucose content (39.74%) of the crude fucoidan was quite similar to the fucose content (43.4%) of the Mediterranean *C. crinita* fucoidan reported by Hadj Ammar et al. [22], but smaller compared to those obtained from other *Cystoseira* sp. (54.5–61.5%) [9]. In addition to fucose and galactose, xylose was among the main constitutive neutral monosaccharides of fucoidan extracted from *C. crinita*. Therefore, crude fucoidan may be qualified as a xylogalactofucan with a Fuc*p*/Gal*p* ratio of 2.56 (Table 2). These results are consistent with earlier studies reported on fucoidans extracted from *A. cribrosum*, *S. vulgare*, *C. costata*, and *S. gurjanovae*, which contained Fuc*p*/Gal*p* ratios of 2.63, 2.15, 2.08, and 3.11, respectively [9,26,30,31].

The monosaccharide profiles of fucoidans are known to vary depending on the extraction method, even within the same seaweed source, because of the heterogeneity of fucoidan structures [32]. Furthermore, the significantly higher fucose and lower xylose and glucose contents of the standard fucoidan sample as compared to the crude fucoidan extract might be attributed to the different processing procedure used in its preparation.

Likewise, it was suggested that the purification process (e.g., alcohol precipitation, dialysis, and ultrafiltration) used to remove the lower molecular weight fractions (LMWF) from the initial extract for isolating only the high-molecular-weight fraction (HMWF) may have helped to reduce the high content of xylose, galactose, and glucose in standard fucoidan polysaccharide. Globally, the monosaccharide compositions of the two fucoidans (crude and standard) were comparable, confirming the presence of sulfated xylogalactofucan polysaccharide structures.

Fucoidans are usually high molecular weight polysaccharides, but medium and low molecular fractions are often also detected [22,23]. The molecular weight of fucoidan can influence the polymer’s biological activity and its therapeutic application. However, an unambiguous relationship between the anti-inflammatory activity and molecular weight of fucoidan is still not established [27]. For example, Park et al. demonstrated that the oral administration of HMWF of fucoidan with Mw = 100 kDa augmented the severity of arthritis and the levels of collagen-specific antibodies, while LMWF with Mw < 30 kDa reduced arthritis through the suppression of Th1-mediated immune reactions [33]. Other authors have proven that *Saccharina longicruris* galactofucan (MW = 638–1529 kDa) reduced fibroblast proliferation, but once depolymerized under 10 kDa, it had no effect on fibroblast cell growth and protein secretion [27,34]. Moreover, some studies reported similar anti-inflammatory effects for high and low-molecular-weight fucoidans. For example, LMWF from *Sargassum hemiphyllum* with Mw = 0.8 kDa and HMWF fraction from *Sargassum horneri* with Mw > 30 kDa both showed, at the same tested dose (100 µg/mL), decreased levels of TNF-α and some interleukines [28,35].

In investigating the anti-inflammatory effects of fucoidan, most of the authors focus on the in vitro effects of this sulfated polysaccharide. Many models of experimental inflammation are described in the literature, and the selection of appropriate pro-inflammatory agents often depends on the stage of the inflammatory response, which is the object of this study. The main mediators during the initial stage of the inflammation are histamine, bradykinin, platelet-activating factor, TNF-α, and prostaglandins. Respectively, the histamine-induced model of inflammation is often used for studying this stage of the response. Histamine also augments the tissue infiltration with inflammatory cells and the following release of nitric oxide (NO), cytokines, and chemokines [36,37].

Our results indicated that fucoidan from *C. crinita* exhibited marked anti-inflammatory activity in histamine-induced rat paw edema. Other studies reported about the same activity for fucoidan from different sources *(Undaria pinnatifida* and *Turbinaria ornata*); however, the polysaccharides were tested on carrageenan-induced paw edema [38,39]. In addition, fucoidan from *Cystoseira sedoides, C. compressa*, and *C. crinita* also reduced the paw inflammation in this model [22]. Moreover, the characteristics of *C. crinita* fucoidan reported by the authors were quite similar to those of the present fucoidan from *C. crinita* (fucose content of 43.4% vs. 41.36%). Manikandan et al. [40] have reported that fucoidan derived from *Turbinaria decurrens* has an anti-inflammatory effect on formalin-induced paw edema in mice.

Anti-inflammatory mechanisms described for fucoidan include the scavenging of free radicals, suppression of the production of nitric oxide, TNF-α, prostaglandin E2, IL-1β, and IL-6 [41]. The observed anti-inflammatory effect of fucoidan could also be related to its high polyphenolic content. The anti-inflammatory activity of the polyphenols was discussed by Mhadhebi et al. [42,43]. The authors have proposed that such effects may be due to polyphenols and sulfated polysaccharides as the main compounds responsible for the antioxidant and anti-inflammatory activity of the evaluated organic and water extracts of seaweeds from the *Cystoseira* genus [42,43].

The polyphenol content, sulfate content, and molecular weight are also involved in the free-radical scavenging activity. Pozharitskaya et al. [44] reported strong antioxidant activity of *F. vesiculosus* fucoidan with the following characteristics: Mw of approximately 735 kDa, neutral carbohydrates of 79.5%, sulfate residues of 27.0%, and uronic acid of 0.7%. The carbohydrates include: fucose (73.5 mol%), glucose (11.8 mol%), galactose (3.7 mol%), xylose (6.6 mol%), mannose (0.2 mol%), and arabinose (0.2 mol%). The authors report the significant inhibition of cyclooxygenase (COX-1 and 2) enzymes, hyaluronidase, and mitogen-activated protein kinase (MAPK) p38 as the main pathways responsible for the registered anti-inflammatory activity. Sulfate content, fucose content, and polyphenols are suggested to contribute to these activities [44]. However, the inflammation is a complex process and is not limited to the formation of free radicals and activation of the COX enzymes. A recent study by Obluchinskaya et al. [45] reported a significant reduction of the protein denaturation and stabilization of human RBC membranes in vitro after treatment with *F. vesiculosus* fucoidan. The authors explained the obtained results with the high fucose and sulfate contents of the studied fucoidan [45].

Jeong et al. [1] reported that the treatment of murine RAW 264.7 macrophages with fucoidan from *Fucus vesiculosus* diminished the secretion of TNF-α and IL-1β in these cells and inhibited the neutrophil infiltration, which revealed its potential to suppress the early stages of the inflammation. Indeed, histamine-induced paw inflammation in rats is a model used primarily for screening anti-inflammatory activity during the early stages of the inflammatory response. Reduced infiltration of the colon tissues with inflammatory cells and decreased submucosal edema are reported by Lean et al. [46] in a model of acute colitis in mice. Oral intake of fucoidan extracts from *Fucus vesiculosus* also significantly lowered the levels of IL-1α, IL-1β, and IL-10 derived from the colon tissues in mice. The anti-inflammatory activity of fucoidan in histamine-induced paw edema is probably related to a decreased release of pro-inflammatory cytokines. In our experiments, we observed decreased serum levels of the pro-inflammatory cytokines TNF-α and IL-1β in rats after treatment with fucoidan from *C. crinita*. Lee et al. [47], Ni et al. [48], and Fernando et al. [49] also reported such a decrease after in vitro treatment with fucoidan fractions derived from *Ecklonia cava*, *Saccharina japonica*, and *Chnoospora minima* on LPS stimulated RAW 264.7 macrophages. Decreased levels of TNF-α, IL-1β, and IL-6 in rats were reported by Aleissa et al. [7]. The authors observed elevated levels of the pro-inflammatory cytokines in a model of streptozotocin-induced diabetes mellitus in rats, and treatment with fucoidan isolated from *Saccharina japonica* reduced these levels. Recent research from Wang et al. (2021) revealed a similar decrease in the pro-inflammatory cytokines after treatment of LPS-stimulated RAW 264.7 macrophages with sulfated polysaccharides from a Celluclast-assisted extract of *Sargassum fulvellum* [50].

Another study performed by Tabarsa et al. [51] evaluated the effect of *Nizamuddinia zanardinii* fucoidan on the RAW264.7 murine macrophage cell line. The authors reported increased secretion of NO, TNF-α, IL-1β, and IL-6 after treatment with fucoidan. Similar results were reported in a recent study by Wang et al. [50] after treatment of the cell culture with sulfated polysaccharides isolated from *Sargassum fulvellum*. Regarding IL-6, we also observed a slight increase in the serum levels, however, the statistical significance margin has not been reached. Probably, the different algal source determines different effects on cytokine levels.

Our study on the levels of the anti-inflammatory cytokine IL-10 showed no significant changes in serum levels after treatment with fucoidan. In contrast, Hwang et al. [28] reported increased levels of this cytokine after in vitro treatment of the Caco-2 cell line. The different results could be related to the molecular weight of the fractions. Hwang et al. [22] tested oligofucoidan with a molecular weight of 0.8 kDa from a different algal source (*Sargassum hemiphyllum*). The role of the molecular weight of fucoidan was discussed by Park et al. [33]. In the study, low-molecular-weight fucoidan fractions from *Undaria pinnatifida* (1 kDa) reduced cartilage and bone destruction and tissue infiltration with inflammatory cells in a model of rheumatoid arthritis in mice, while high-molecular-weight fractions had the opposite effect.

The importance of the algal source was reported by other authors in a model of chronic colitis in mice. Fucoidan derived from *Cladosiphon okamuranus* Tokida decreased both IFN-γ and IL-6 synthesis and increased levels of IL-10 in the lamina propria of the colon, while fucoidan from *Fucus vesiculosus* did not induce changes in the levels of these cytokines [52]. However, another important factor for the activity of fucoidan is the molecular weight. Low molecular weight (LMW) and high molecular weight (HMW) fucoidan could have opposite effects, as reported by Park et al. [33]. The fucoidan isolated from *C. crinita* consists of two fractions and could be classified as LMW fucoidan. Recent research by Wu et al. [53] showed the anti-inflammatory activity of fucoidan from *L. japonica* and similar characteristics to *C. crinita* fucoidan (Mw 8.1 kDa and high fucose content). The authors found decreased levels of TNF-α and IL-6 in lung tissues after treatment with bleomycine. Chen et al. [54] also reported decreased synthesis of TNF-α in LPS-treated Hep-G cells after treatment with LMW *S. siliquosum* fucoidan (3kDa).

*L*-fucose is found to decrease elevated levels of TNF-α, IL-1β, and IL-6 in serum and colonic tissues of mice with a model of colitis [55]. The anti-inflammatory effect and the changes in the cytokine levels in the current study could be partially related to the high fucose content of the isolated fucoidan. Pozharitskaya et al. (2020) also demonstrated the anti-inflammatory activity of fucoidan with high fucose content (79.5% neutral carbohydrates represented by 73.5 mol% fucose [44].

We evaluated the effects of *C. crinita* fucoidan using the intraperitoneal route of application. However, other routes could also be found suitable for this sulfated polysaccharide. Fucoidan isolated from *Fucus vesiculosus* has shown good skin penetrating properties after topical application in rats, and cream formulations containing the same fucoidan reduced carrageenan-induced allodynia in rats [41,56]. Low- (7.6 kDa) and medium-MW (35 kDa) fucoidans from *Laminaria japonica* also showed good absorption after oral administration to rats [57].

No significant changes in the levels of TNF-α were observed after a single dose of *C. crinita* fucoidan in rats with carrageenan-induced peritonitis. Fucoidan from *Cladosiphon okamuranus* decreased the neutrophil infiltration of the peritoneal cavity in a model of acute peritonitis in rats, as reported by Cumashi et al. [58]. However, the levels of pro-inflammatory cytokines in the peritoneal fluid have not been determined. To our knowledge, this is the first study that reports an evaluation of these levels in the peritoneal fluid.

## 4. Materials and Methods

### 4.1. Algae Material and Chemicals

**Figure 8:**
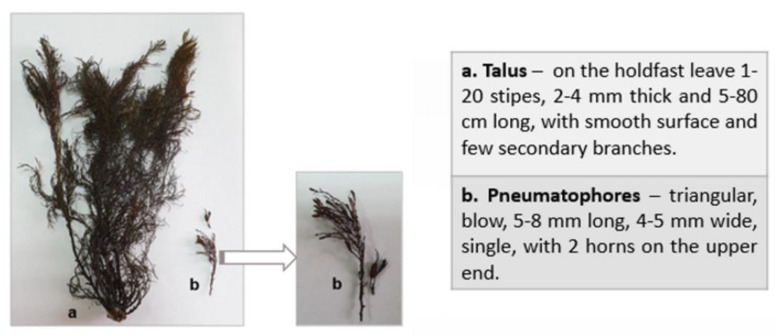
Macrosopic identification of *Cystoseira crinita* (Desf.) Bory.

Fucoidan was isolated from *Cystoseira crinita* (Desf.) Bory, collected near Arapya beach, the Black Sea region, Bulgaria (42°11′17.9′′ N, 27°50’20.0” E), in July 2019. The taxonomic identification of algae species was performed on the basis of diagnostic macroscopic features, with the assistance of the Institute of Oceanology “Fridtjof Nansen” and the Department of Pharmaceutical Botany, Medical University-Plovdiv (assoc. prof. Plamen Stoyanov, PhD) (Figure 8).

The following solutions for injection were purchased from a pharmacy store and used:

1. Diclofenac sodium (Almiral^®^, Limassol, Cyprus)–manufacturer: Medochemie; batch number: A902B0; expiration date: 09.2023; excipients: benzyl alcohol, sodium formaldehyde sulfoxylate, propylene glycol, sodium metabisulfite, sodium hydroxide, and water for injections.
2. Dexamethasone phosphate (Dexamethason KRKA^®^, Novo Mesto, Slovenia)-manufacturer: KRKA; batch number: A67892; expiration date: 30 March 2023; excipients: glycerol, disodium EDTA, sodium phosphate dihydrate, water for injections.
3. Heparin sodium (Heparinum WZF^®^, Warsaw, Poland)–manufacturer: Warsaw Pharmaceutical Works Polfa S.A., Poland; batch number: 01BK1219; expiration date: 12.2022; excipients: NaCl, Benzyl alcohol, 10% NaOH, water for injection.

Fucoidan from *Fucus vesiculosus* (Product No. F5631; Batch No. SLBC4004V), lipopolysaccharides from *Escherichia coli* O55:B5 (LPS), histamine, and all other reagents were obtained from Sigma-Aldrich and were of analytical grade. All tested fucoidans (from *Fucus vesiculosus* and *C. crinita),* histamine, and λ-carrageenan were dissolved in saline on the day of each experiment.

### 4.2. Animals

Male Wistar rats with an average weight of 170–270 g were used. Animals were housed under standard laboratory conditions: temperature 22 ± 1 °C, humidity 45%, a 12:12 h light/dark cycle, food, and water ad libitum.

### 4.3. Extraction of Fucoidan

**Figure 9:**
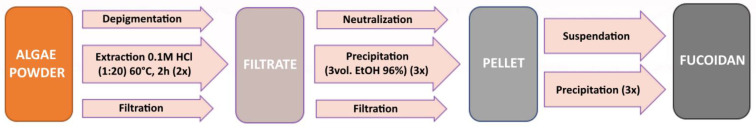
Extraction process of fucoidan from *C. crinita*.

The collected fresh algae were cleaned of available epiphytes, washed with tap water, and dried in the sun at an average daily temperature of 35 °C until a constant weight was obtained. Prior to the extraction process, the algae were treated with an ethanol:chloroform:water solution (80:5:15, *v/v/v*) to remove pigments, lipids, and phenolic substances [59]. Then, the extraction and separation of fucoidan followed the protocol proposed by Hentati et al. [9], with slight modifications. Dried algae mass was treated twice with 0.1 M HCl (algae:solvent ratio 1:20, *w/v*) during 2 h at 60 °C with continuous stirring (650 rpm). The obtained extract was separated by centrifugation (40 min, 5000 rpm, 4 °C) and filtered through a glass filter (16–40 μm). The filtrate was then neutralized to pH 7.5 with 3 M NaOH, concentrated, and precipitated three times with three volumes of 96% ethanol (−20 °C). Subsequently, the supernatant was removed by centrifugation (15 min, 5000 rpm, 4 °C), and the pellet was suspended in water (30 g/L) for 12 h, precipitated with ethanol, and finally dried at 50 °C using a drying oven (Figure 9).

### 4.4. Chemical Content of Crude Fucoidan from C. crinita

Previously to the analyses, the fucoidan polysaccharides were dissolved in distilled water at a concentration of 10 g/L. The amount of neutral sugars was determined by the phenol-sulfuric acid method of Dubois et al. [60] using glucose as a standard (20–100 μg/mL). Uronic acid content was estimated following the protocol of Blumenkrantz & Asboe-Hansen [61] using H_2_SO_4_/tetraborate and a standard of glucuronic acid (25–150 μg/mL). Sulfate content was carried out by Dogson and Price [62] methodology using K_2_SO_4_ and BaCl_2_. Phenolic compounds were estimated by the method of Singleton and Rossi [63] using the Folin–Ciocalteu reagent and gallic acid as a standard (0–20 μg/mL). A protein assay was carried out by the Bradford method [64] calibrated against bovine serum albumin (0–100 μg/mL). All measurements were performed on a Beckman Coulter DU 800 spectrophotometer, Brea, CA, USA.

### 4.5. Monosaccharide Composition

Prior to analysis, the fucoidan polysaccharides (2.5 mg) were hydrolyzed at 100 °C for 4 h using 4 M TFA in a sealed 8 mL Pyrex glass screw-cap tube, and the remaining TFA was evaporated to dryness at 30 °C in a speed-vac centrifuge under low pressure. The dried samples were dissolved in 1 mL of Milli-Q water and analyzed by HPAEC-PAD according to Boucelkha et al. [11].

HPAEC-PAD analyses were performed on a Dionex ICS-3000 system (Dionex Corp., Sunnyvale, CA, USA) consisting of an SP gradient pump, an AS autosampler, an ED electrochemical detector with a gold working electrode, an Ag/AgCl reference electrode, and Chromeleon version 6.5 (Dionex Corp., Sunnyvale, CA, USA). A Carbopac PA1 (4 × 250 mm, Dionex) column with a guard column (4 × 50 mm, Dionex) was used as a stationary phase, using different eluents depending on the nature of the monosaccharides being analyzed. Two eluents were used for effective uronic acid separation: eluent A (100 mM NaOH) and eluent B (100 mM NaOH and 1 M NaOAc). The two eluents were mixed to form the following gradient (% of B in A): t = 0 min: 0%; from 0 to 60 min: linear gradient of 0 to 100%. After each run, the column was washed for 10 min with 100% B and preequilibrated for 15 min with the starting conditions of the employed gradient. Samples (2.5 mg/mL) were injected through a 25 μL full loop, and separations were performed at a rate of 1 mL/min.

The neutral monosaccharides were eluted isocratically with 16 mM NaOH at a flow rate of 1 mL/min. Each neutral monosaccharide concentration was determined after integration of the respective areas and compared with standard curves obtained with rhamnose, arabinose, mannose, galactose, xylose, glucose, and fucose (Sigma-Aldrich). For eluent preparation, Milli-Q water and 50% (*w/v*) NaOH and NaOAc were used. All eluents were degassed before use by flushing helium through for 30 min; subsequently, they were kept under a constant helium pressure (eluent degassing module, Dionex).

### 4.6. FTIR Spectroscopy

Fourier-transform infrared (FTIR) measurements were carried out using a Nicolet iS 10 FTIR spectrometer (Thermo Fisher Scientific, Pittsburgh, PA, USA), equipped with a diamond attenuated total reflection (ATR) accessory. The IR spectra (64 scans) were recorded at room temperature (referenced against air) with a wavenumber range of 650–4000 cm^−1^ and a resolution of 4 nm.

### 4.7. H NMR Analysis

The freeze-dried samples were dissolved in D2O at 10–15 g/L. ^1^H NMR spectrum was recorded at 80 °C on a Bruker Avance 500 MHz spectrometer operating at 500.08 MHz for 1H, using a multinuclear probe BBI 5 mm. A 1D proton with a water suppression pulse sequence (NOESY 1D) was acquired. The sequence repeat was –D1-t-90°-t-90°-tm-90°-AQ, where D1 (10 s) is the relaxation delay, 90° is the already determined 90° radio-frequency pulse length, t (9.49 μs) is a very short delay, tm (0.15 s) is a mixing time delay, and AQ (5.45 s) is the data acquisition time. Low-power rf irradiation was applied at the water frequency during D1 and tm to presaturate the water signal. The spectrum was acquired using 256 scans of 64 K data points with spectral widths of 6009.62 Hz. The resulting 1H spectrum was manually phased, baseline-corrected, and calibrated to TMSP (TriMethyl Silyl propionate) at 0 ppm, all using TopSpin 3.6 (BRUKER BioSpin, Rheinstetten, Germany) [11].

### 4.8. SEC-MALLs Analysis

The molecular weight of the sulfated fucoidan polysaccharides was determined by size-exclusion chromatography (SEC) equipped with multi-angle light scattering (MALS). The SEC line consisted of an SB-G guard column as protection and three columns in series (SB-806 HQ, SB-804 HQ, and SB-803 HQ, 300 mm L × 8 mm I.D., Shodex Showa Denko K.K., Tokyo, Japan). The elution was performed at a flow rate of 0.5 mL/min (LC-20AD, Shimadzu, Duisburg, Germany). NaNO_3_, 0.1 M, and NaN_3_, 2.5 mM, used as carriers, were filtered through a 0.02 µm, 47 mm membrane filter (Anotop 47, Whatman, Maidstone, UK), and carefully degassed. Samples (2.5 mg/mL) were filtered through a 0.45 µm membrane filter (Grace Altech, Darmstadt, Germany) and were injected through a 100 µL full loop. Detection was achieved with a light scattering detector (MiniDAWN TREOS II, Wyatt Technology Corporation, Santa Barbara, CA, USA) and a refractive index detector (RID-10 A, Shimadzu, Duisburg, Germany). Data acquisition and processing were performed using ASTRA 7.2.2 software. Specific refractive index increments (dn/dc) of 0.150 were used according to the literature.

### 4.9. Histamine-Induced Paw Edema

Forty male Wistar rats (weight 170–210 g) were divided into five groups (n = 8) and treated intraperitoneally as follows: 1st group (control)—treated with saline (0.1 mL/100 g bw), 2nd group (diclofenac)—treated with diclofenac sodium in a dose of 25 mg/kg bw, 3rd group (fucoidan standard)—treated with 50 mg/kg bw fucoidan from *Fucus vesiculosus*, 4th group (fucoidan 25 mg/kg)—treated with 25 mg/kg bw fucoidan from *C. crinita*, and 5th group (fucoidan 50 mg/kg)—treated with 50 mg/kg bw fucoidan from *C. crinita*. The volume of each injection was 0.1 mL/100g bw. One hour after the treatment, the animals received a subplantar injection of 0.1 mL of a 0.1% solution of histamine in saline into the right paw [65]. Before the injection of histamine and 5, 15, 30, 60, 90, and 120 min after it, the anti-inflammatory effect was measured using a plethysmometer (UgoBasile, Gemonio, Italy), as described previously [66].

The paw edema was calculated according to the formula:

(1) $$\mathrm{Percentage}\;\mathrm{of}\;\mathrm{increase}\;(\%)=\frac{V_{n}-V_{0}}{V_{0}}\times 100$$

*V_n_* = the volume of the right hind paw measured after carrageenan injection at the n minute;

*V*_0_ = the volume of the right hind paw measured for the same animal before histamine injection.

### 4.10. Detection of Immunomodulatory Cytokines

The experimental protocol and the tested groups were designed according to the articles by Kostadinov et al. [67] and Ohgy et al. [68]. Twenty-four male Wistar rats (with a weight of 170–270 g) were divided into three groups (n = 8) and treated intraperitoneally as follows: 1st group (control)—treated with saline (0.1 mL/100 g bw), 2nd group (fucoidan 25 mg/kg)—treated with 25 mg/kg bw fucoidan from *C. crinita*, and 3rd group (fucoidan 50 mg/kg)—treated with 50 mg/kg bw fucoidan from *C. crinita*. Thirty minutes after the application, a solution of LPS in saline was injected intraperitoneally at a dose of 0.25 mg/kg. Four hours after the second injection, the rats were sacrificed, and blood samples were collected in monovettes. The monovettes were transported immediately to the Department of Microbiology in an ice container.

In the Department of Microbiology and Immunology, blood samples and peritoneal fluids were immediately centrifuged at 1000× *g* for 10 min at room temperature. The supernatants were subsequently achieved, aliquoted (250–500 μL) to avoid repeated freeze-thaw cycles, and stored at −80 °C until use. The serum concentrations of TNF-α, IL-1β, IL-6 and IL-10 and TNF-α concentrations in peritoneal fluid were measured by a specific enzyme-linked immunosorbent assay (ELISA) using pre-coated strip plates. The tests were performed using the Rat IL-6 ELISA KIT of Diaclone (CEDEX—Besançon, Franche-Comté, France), Rat TNF-α ELISA KIT of Diaclone (CEDEX—Besançon, Franche-Comté, France), Rat IL-1β ELISA KIT of Diaclone (CEDEX—Besançon, Franche-Comté, France), and Rat IL-10 ELISA KIT of Diaclone (CEDEX—Besançon, Franche-Comté, France), strictly following the manufacturer’s recommendations. The optical density was detected at 450 nm with an optional 620 nm reference filter using the Tecan Sunrise Microplate Reader (Tecan Austria GmbH, Groedig, Salzburg,) and Magellan™ Data Analysis Software (Tecan Trading AG, V 7.2., Männedorf, Switzerland). Each sample concentration was calculated from the linear equation derived from the standard curve of the concentrations of the cytokine. The concentrations of inflammatory factors were presented as picograms per milliliter (pg/mL).

### 4.11. Carrageenan-Induced Model of Peritonitis

The experiment was performed as described by de Carvalho et al. [69]. Twenty-four male Wistar rats (weight 170–260 g) were divided into three groups (n = 8) and treated intraperitoneally as follows: 1st group (control)—treated with saline (0.1 mL/100 g bw), 2nd group (dexamethasone)—treated with dexamethasone phosphate at a dose of 0.2 mg/kg bw, and 3rd group (fucoidan)—treated with 25 mg/kg bw fucoidan from *C. crinita*. One hour later, a solution of λ-carrageenan in saline (0.5 mg/mL; 1 mL) was injected intraperitoneally. Four hours after the second injection, the rats were sacrificed, and peritoneal fluid was obtained after washing the peritoneal cavity with a solution containing 50 UI of heparin and 10 mL saline. The abdominal part of the rats was massaged gently, and a volume of 5 mL peritoneal fluid was obtained from each animal. The monovettes containing the fluid were transported immediately in an ice container to the Department of Microbiology and Immunology.

### 4.12. Statistical Analysis

Statistical analysis was performed using SPSS 17.0. The normal distribution was evaluated with a one-sample Kolmogorov–Smirnov test. A one-way ANOVA and Bonferroni post hoc test were employed for further analysis. The number of tested animals is given as *n*. The results are presented as mean ± SEM and are considered significant at *p* < 0.05.

## 5. Conclusions

Fucoidan from *C. crinita* showed a well-defined anti-inflammatory effect in the histamine-induced model of paw inflammation in rats. This sulfated polysaccharide also attenuated the levels of some pro-inflammatory cytokines (TNF-α and IL-1β) in rat blood serum after LPS challenge, while changes in the anti-inflammatory cytokine IL-10 were not observed. The decreased levels of pro-inflammatory cytokines, the low Mw, and the chemical composition of *C. crinita* fucoidan may provide an explanation for the anti-phlogistic activity of the sulfated polysaccharide. Finally of note to mention is that further study should be conducted in the future to highlight the mechanisms of this *C. crinita* fucoidan involved in the anti-inflammatory reaction.

## Figures and Tables

**Table 1:** Extraction yield and chemical content of *C. crinita* crude fucoidan.

Sample	Extraction Yield (%)	Neutral Sugars (%, *w/w*)	Uronic Acid (%, *w/w*)	Sulfates (%, *w/w*)	Total Polyphenols (%)	Protein (%)
*C. crinita* crude fucoidan	5.15 ± 0.62	46.64 ± 2.58	13.15 ± 0.34	17.00 ± 2.00	<0.10	0.56 ± 0.05

**Table 2:** Monosaccharide composition (molar percentage) of fucoidan extracted from *C. crinita* (crude fucoidan) and fucoidan standard from *F. vesiculosus* (standard fucoidan).

Molar Percentage (%)							
Samples	Fucose	Rhamnose	Arabinose	Galactose	Glucose	Xylose	Glucuronic Acid
Crude fucoidan	39.74 ± 0.15	2.37 ± 0.11	2.13 ± 0.12	15.51 ± 0.12	5.50 ± 0.08	20.75 ± 0.22	13.52 ± 0.11
Standard fucoidan	55.69 ± 1.47	2.04 ± 0.52	0.81 ± 0.03	13.40 ± 1.27	1.20 ± 0.06	14.71 ± 0.14	11.41 ± 0.44

**Table 3:** Average macromolecular characteristics of *C. crinita* crude fucoidan determined by SEC/MALS analysis.

Peak Limit (mL)	Mn (g/mol)	Mw (g/mol)	Polydispersity (Mw/Mn)
19.86–27.63	2.26 × 10^4^	1.24 × 10^5^	5.45
19.86–21.88	4.23 × 10^5^	5.34 × 10^5^	1.26
21.88–25.47	5.36 × 10^4^	7.01 × 10^4^	1.31
25.47–27.63	1.17 × 10^4^	1.38 × 10^4^	1.18

**Table 4:** Mean percentages of increase in the rat paw volume in a model of histamine-induced edema after treatment with saline (controls), diclofenac sodium (diclofenac 25 mg/kg), fucoidan standard from *F. vesiculosus* (fucoidan standard 50 mg/kg), and fucoidan test from *C. crinita* in two doses (fucoidan test 25 mg/kg and fucoidan test 50 mg/kg), respectively.

	Time Point	5 min	15 min	30 min	60 min	90 min	120 min
Groups							
Controls	Mean	31.94	50.72	59.94	52.32	43.44	38.58
	SEM	2.23	4.05	3.85	2.98	4.52	5.03
diclofenac 25 mg/kg	Mean	12.57 **	19.25 ***	20.48 ***	18.05 ***	15.24 ***	12.90 ***
	SEM	1.73	1.50	2.13	1.91	2.40	2.22
fucoidan standard 50 mg/kg	Mean	29.28 ^#^	39.34 ^#^	45.58 ^##^	34.27 ^#^**	28.18 *	20.65 **
	SEM	4.61	7.72	7.82	4.90	5.51	4.31
fucoidan test 25 mg/kg	Mean	8.08 ***^†^	10.36 ***^†††^	13.20 ***^†††^	7.49 ***^†††^	4.21 ***^†††^	2.85 ***^††^
	SEM	1.41	2.77	4.24	3.43	1.93	1.28
fucoidan test 50 mg/kg	Mean	16.28 *	16.52 ***^†^	16.06 ***^††^	9.94 ***^†††^	8.06 ***^††^	3.45 ***^††^
	SEM	3.09	3.49	3.56	2.83	2.10	1.26

* *p* < 0.05 vs. controls at the same time; ** *p* < 0.01 vs. controls at the same time; *** *p* < 0.001 vs. controls at the same time; ^#^ *p* < 0.05 vs. diclofenac at the same time; ^##^ *p* < 0.01 vs. diclofenac at the same time; ^†^ *p* < 0.05 vs. fucoidan standard at the same time; ^††^ *p* < 0.01 vs. fucoidan standard at the same time; ^†††^ *p* < 0.001 vs. fucoidan standard at the same time.

## Data Availability

The data presented in this study are available on request from the corresponding author.

## References

[1] Jeong J.W., Hwang S.J., Han M.H., Lee D.S., Yoo J.S., Choi I.W., Cha H.J., Kim S., Kim H.S., Kim G.Y. (2017). Fucoidan inhibits lipopolysaccharide-induced inflammatory responses in RAW 264.7 macrophages and zebrafish larvae. Mol. Cell. Toxicol..

[2] Muralidharan S., Mandrekar P. (2013). Cellular stress response and innate immune signaling: Integrating pathways in host defense and inflammation. J. Leukoc. Biol..

[3] Kennedy M.A. (2010). A brief review of the basics of immunology: The innate and adaptive response. Vet. Clin. N. Am. Small Anim. Pract..

[4] Kyung J., Kim D., Park D., Yang Y.H., Choi E.K., Lee S.P., Kim T.S., Lee Y.B., Kim Y.B. (2012). Synergistic anti-inflammatory effects of *Laminaria japonica* fucoidan and *Cistanche tubulosa* extract. Lab. Anim. Res..

[5] Wang Y., Xing M., Cao Q., Ji A., Liang H., Song S. (2019). Biological activities of fucoidan and the factors mediating its therapeutic effects: A review of recent studies. Mar. Drugs.

[6] Ale M.T., Meyer A.S. (2013). Fucoidans from brown seaweeds: An update on structures, extraction techniques and use of enzymes as tools for structural elucidation. RSC Adv..

[7] Aleissa M.S., Alkahtani S., Abd Eldaim M.A., Ahmed A.M., Bungău S.G., Almutairi B., Bin-Jumah M., AlKahtane A.A., Alyousif M.S., Abdel-Daim M.M. (2020). Fucoidan ameliorates oxidative stress, inflammation, DNA damage, and hepatorenal injuries in diabetic rats intoxicated with aflatoxin B1. Oxid. Med. Cell. Longev..

[8] Myers S.P., Mulder A.M., Baker D.G., Robinson S.R., Rolfe M.I., Brooks L., Fitton J.H. (2016). Effects of fucoidan from *Fucus vesiculosus* in reducing symptoms of osteoarthritis: A randomized placebo-controlled trial. Biologics.

[9] Hentati F., Delattre C., Ursu A.V., Desbrières J., Le Cerf D., Gardarin C., Abdelkafi S., Michaud P., Pierre G. (2018). Structural characterization and antioxidant activity of water-soluble polysaccharides from the Tunisian brown seaweed *Cystoseira compressa*. Carbohydr. Polym..

[10] Bouissil S., Alaoui-Talibi Z.E., Pierre G., Rchid H., Michaud P., Delattre C., El Modafar C. (2020). Fucoidans of Moroccan brown seaweed as elicitors of natural defenses in palm roots. Mar. Drugs.

[11] Boucelkha A., Petit E., Elboutachfaiti R., Molinié R., Amari S., Yahaoui R.Z. (2017). Production of guluronate oligosaccharide of alginate from brown algae *Stypocaulon scoparium* using an alginate lyase. J. Appl. Phycol..

[12] Zhang Z., Khan N.M., Nunez K.M., Chess E.K., Szabo C.M. (2012). Complete monosaccharide analysis by high-performance anion-exchange chromatography with pulsed amperometric detection. Anal. Chem..

[13] Wijesingh S., Benslima A., Barragan-Montero V., Hajji M., Nasri M. (2017). Polyphenolic-protein-polysaccharide ternary conjugates from *Cystoseira barbata* Tunisian seaweed as potential biopreservatives: Chemical, antioxidant and antimicrobial properties. Int. J. Biol. Macromol..

[14] Dammak M., Hadrich B., Miladi R., Barkallah M., Hentati F., Hachicha R., Laroche C., Michaud P., Fendri I., Abdelkafi S. (2017). Effects of nutritional conditions on growth and biochemical composition of *Tetraselmis* sp. Lipids Health Dis..

[15] Ermakova S., Men’shova R., Vishchuk O., Kim S.M., Um B.H., Isakov V., Zvyagintseva T. (2013). Water-soluble polysaccharides from the brown alga *Eisenia bicyclis*: Structurl characteristics and antitumor activity. Algal Res..

[16] Sellimi S., Kadri N., Barragan-Montero V., Laouer H., Hajji M., Nasri M. (2014). Fucans from a Tunisian brown seaweed *Cystoseira barbata*: Structural characteristics and antioxidant activity. Int. J. Biol. Macromol..

[17] Chevolot L., Foucault A., Chaubet F., Kervarec N., Sinquin C., Fisher A.M., Boisson-Vidal C. (1999). Further data on the structure of brown seaweed fucans: Relationships with anticoagulant activity. Carbohydr. Res..

[18] Alves A.P., Mulloy B., Diniz J.A., Mourão P.A. (1997). Sulfated polysaccharides from the egg jelly layer are species-specific inducers of acrosomal reaction in sperms of sea urchins. J. Biol. Chem..

[19] Mishra A., Kavita K., Jha B. (2011). Characterization of extracellular polymeric substances produced by micro-algae *Dunaliella salina*. Carbohydr. Polym..

[20] Ailiesei G.L., Ciobanu M., Balan M., Stavarache C., Barbes L., Nicolescu A., Deleanu C. (2015). NMR detected metabolites in complex natural fluids. Quinic acid in apple juice. Ovidius Univ. Ann. Chem..

[21] Gong P.X., Wu Y.C., Liu Y., Lv S.Z., You Y., Zhou Z.L., Chen X., Li H.J. (2022). Structure and hypoglycemic effect of a neutral polysaccharide isolated from sea cucumber *Stichopus japonicus*. Int. J. Biol. Macromol..

[22] Hadj Ammar H., Lajili S., Ben Said R., Le Cerf D., Bouraoui A., Majdoub H. (2015). Physico-chemical characterization and pharmacological evaluation of sulfated polysaccharides from three species of Mediterranean brown algae of the genus *Cystoseira*. DARU.

[23] Zayed A., El-Aasr M., Ibrahim A.R.S., Ulber R. (2020). Fucoidan characterization: Determination of purity and physicochemical and chemical properties. Mar. Drugs.

[24] Apostolova E., Lukova P., Baldzhieva A., Katsarov P., Nikolova M., Iliev I., Peychev L., Trica B., Oancea F., Delattre C. (2020). Immunomodulatory and anti-inflammatory effects of fucoidan: A review. Polymers.

[25] Hahn T., Lang S., Ulber R., Muffler K. (2012). Novel procedures for the extraction of fucoidan from brown algae. Process Biochem..

[26] Prokofjeva M., Imbs T., Shevchenko N., Spirin P., Horn S., Fehse B., Zvyagintseva T., Prassolov V. (2013). Fucoidans as potential inhibitors of HIV-1. Mar. Drugs.

[27] Flórez-Fernández N., Torres M.D., González-Muñoz M.J., Domínguez H. (2018). Potential of intensification techniques for the extraction and depolymerization of fucoidan. Algal Res..

[28] Hwang P.A., Phan N.N., Lu W.J., Ngoc Hieu B.T., Lin Y.C. (2016). Low-molecular-weight fucoidan and high-stability fucoxanthin from brown seaweed exert prebiotics and anti-inflammatory activities in Caco-2 cells. Food Nutr. Res..

[29] Li B., Lu F., Wei X., Zhao R. (2008). Fucoidan: Structure and bioactivity. Molecules.

[30] Cho T.M., Kim W.J., Moon S.K. (2014). AKT signaling is involved in fucoidan-induced inhibition of growth and migration of human bladder cancer cells. Food Chem. Toxicol..

[31] Dore C.M.P.G., Alves M.G.D.C.F., Will L.S.E.P., Costa T.G., Sabry D.A., de Souza Rêgo L.A.R., Accardo C.M., Rocha H.A.O., Filgueira L.G.A., Leite E.L. (2013). A sulfated polysaccharide, fucans, isolated from brown algae *Sargassum vulgare* with anticoagulant, antithrombotic, antioxidant and anti-inflammatory effects. Carbohydr. Polym..

[32] Wijesinghe W.A.J.P., Jeon Y.J. (2012). Biological activities and potential industrial applications of fucose rich sulfated polysaccharides and fucoidans isolated from brown seaweeds: A review. Carbohydr. Polym..

[33] Park S.B., Chun K.R., Kim J.K., Suk K., Jung Y.M., Lee W.H. (2010). The differential effect of high and low molecular weight fucoidans on the severity of collagen-induced arthritis in mice. Phytother. Res..

[34] Rioux L.E., Moulin V., Beaulieu M., Turgeon S.L. (2013). Human skin fibroblast response is differentially regulated by galactofucan and low molecular weight galactofucan. Bioact. Carbohydr. Diet. Fibre.

[35] Sanjeewa K.A., Fernando I.P.S., Kim E.A., Ahn G., Jee Y., Jeon Y.J., Noh H.J., Koh H.B., Kim H.K., Cho H.H. (2017). Anti-inflammatory activity of a sulfated polysaccharide isolated from an enzymatic digest of brown seaweed *Sargassum horneri* in RAW 264.7 cells. Nutr. Res. Pract..

[36] Morris C.E., Skalak T.C. (2008). Acute exposure to a moderate strength staticmagnetic field reduces edema formation in rats. Am. J. Physiol. Heart Circ. Physiol..

[37] Draganova-Filipova M., Apostolova E., Zagorchev P. (2018). Effects of *Rosmarinus officinalis* oil on histamine-induced acute inflammation. Compt. Rend. Acad. Bulg. Sci..

[38] Phull A.R., Kim S.J. (2017). Fucoidan as bio-functional molecule: Insights into the anti-inflammatory potential and associated molecular mechanisms. J. Funct. Foods.

[39] Ananthi S., Raghavendran H.R., Sunil A.G., Gayathri V., Ramakrishnan G., Vasanthi H.R. (2010). *In vitro* antioxidant and *in vivo* anti-inflammatory potential of crude polysaccharide from *Turbinaria ornata* (Marine Brown Alga). Food Chem. Toxicol..

[40] Manikandan R., Parimalanandhini D., Mahalakshmi K., Beulaja M., Arumugam M., Janarthanan S., Palanisamy S., You S., Prabhu N.M. (2020). Studies on isolation, characterization of fucoidan from brown algae *Turbinaria decurrens* and evaluation of it’s in vivo and in vitro anti-inflammatory activities. Int. J. Biol. Macromol..

[41] Obluchinskaya E.D., Pozharitskaya O.N., Flisyuk E.V., Shikov A.N. (2021). Formulation, optimization and in vivo evaluation of fucoidan-based cream with anti-inflammatory properties. Mar. Drugs.

[42] Mhadhebi L., Laroche-Clary A., Robert J., Bouraoui A. (2011). Antioxidant, anti-inflammatory, and antiproliferative activities of organic fractions from the Mediterranean brown seaweed Cystoseira sedoides. Can. J. Physiol. Pharmacol..

[43] Mhadhebi L., Mhadhebi A., Robert J., Bouraoui A. (2014). Antioxidant, anti-inflammatory and antiproliferative effects of aqueous extracts of three mediterranean brown seaweeds of the genus cystoseira. IJPR.

[44] Pozharitskaya O.N., Obluchinskaya E.D., Shikov A.N. (2020). Mechanisms of bioactivities of fucoidan from the brown seaweed *Fucus vesiculosus* L. of the Barents Sea. Mar. Drugs.

[45] Obluchinskaya E.D., Pozharitskaya O.N., Shikov A.N. (2022). In vitro anti-inflammatory activities offFucoidans from five species of brown seaweeds. Mar. Drugs.

[46] Lean Q.Y., Eri R.D., Fitton J.H., Patel R.P., Gueven N. (2015). Fucoidan extracts ameliorate acute colitis. PLoS ONE.

[47] Lee S.H., Ko C.I., Ahn G., You S., Kim J.S., Heu M.S., Kim J., Jee Y., Jeon Y.J. (2012). Molecular characteristics and anti-inflammatory activity of the fucoidan extracted from *Ecklonia cava*. Carbohydr. Polym..

[48] Ni L., Wang L., Fu X., Duan D., Jeon Y.J., Xu J., Gao X. (2020). *In vitro* and *in vivo* anti-inflammatory activities of a fucose-rich fucoidan isolated from *Saccharina japonica*. Int. J. Biol. Macromol..

[49] Fernando I.S., Sanjeewa K.A., Samarakoon K.W., Lee W.W., Kim H.S., Kang N., Ranasinghe P., Lee H.S., Jeon Y.J. (2017). A fucoidan fraction purified from *Chnoospora minima*; a potential inhibitor of LPS-induced inflammatory responses. Int. J. Biol. Macromol..

[50] Wang L., Yang H.-W., Ahn G., Fu X., Xu J., Gao X., Jeon Y.-J. (2021). In Vitro and in vivo anti-inflammatory effects of sulfated polysaccharides isolated from the edible brown seaweed, Sargassum fulvellum. Mar. Drugs.

[51] Tabarsa M., Dabaghian E.H., You S., Yelithao K., Cao R., Rezaei M., Alboofetileh M., Bita S. (2020). The activation of NF-κB and MAPKs signaling pathways of RAW264.7 murine macrophages and natural killer cells by fucoidan from *Nizamuddinia zanardinii*. Int. J. Biol. Macromol..

[52] Matsumoto S., Nagaoka M., Hara T., Kimura-Takagi I., Mistuyama K., Ueyama S. (2004). Fucoidan derived from *Cladosiphon okamuranus* Tokida ameliorates murine chronic colitis through the down-regulation of interleukin 6 production on colonic epithelial cells. Clin. Exp. Immunol..

[53] Wu N., Li Z., Wang J., Geng L., Yue Y., Deng Z., Wang Q., Zhang Q. (2021). Low molecular weight fucoidan attenuating pulmonary fibrosis by relieving inflammatory reaction and progression of epithelial-mesenchymal transition. Carbohydr. Polym..

[54] Chen C.Y., Wang S.H., Huang C.Y., Dong C.D., Huang C.Y., Chang C.C., Chang J.S. (2021). Effect of molecular mass and sulfate content of fucoidan from Sargassum siliquosum on antioxidant, anti-lipogenesis, and anti-inflammatory activity. J. Biosci. Bioeng..

[55] He R., Li Y., Han C., Lin R., Qian W., Hou X. (2019). *L*-Fucose ameliorates DSS-induced acute colitis via inhibiting macrophage M1 polarization and inhibiting NLRP3 inflammasome and NF-kB activation. Int. Immunopharmacol..

[56] Pozharitskaya O.N., Shikov A.N., Obluchinskaya E.D., Vuorela H. (2019). The pharmacokinetics of fucoidan after topical application to rats. Mar. Drugs.

[57] Shikov A.N., Flisyuk E.V., Obluchinskaya E.D., Pozharitskaya O.N. (2020). Pharmacokinetics of marine-derived drugs. Mar. Drugs.

[58] Cumashi A., Ushakova N.A., Preobrazhenskaya M.E., D’incecco A., Piccoli A., Totani L., Tinari N., Morozevich G.E., Berman A.E., Bilan M.I. (2007). A comparative study of the anti-inflammatory, anticoagulant, antiangiogenic, and antiadhesive activities of nine different fucoidans from brown seaweeds. Glycobiology.

[59] Zayed A., Muffler K., Hahn T., Rupp S., Finkelmeier D., Burger-Kentischer A., Ulber R. (2016). Physicochemical and biological characterization of fucoidan from *Fucus vesiculosus* purified by dye affinity chromatography. Mar. Drugs.

[60] Dubois M., Gilles K., Hamilton J.K., Rebers P.A., Smith F. (1951). A colorimetric method for the determination of sugars. Nature.

[61] Blumenkrantz N., Asboe-Hansen G. (1973). New method for quantitative determination of uronic acids. Anal. Biochem..

[62] Dodgson K.S., Price R.G. (1962). A note on the determination of the ester sulphate content of sulphated polysaccharides. Biochem. J..

[63] Singleton V.L., Orthofer R., Lamuela-Raventós R.M. (1999). Analysis of total phenols and other oxidation substrates and antioxidants by means of Folin-Ciocalteu reagent. Method. Enzymol..

[64] Bradford M.M. (1976). A rapid and sensitive method for the quantitation of microgram quantities of protein utilizing the principle of protein-dye binding. Anal. Biochem..

[65] Nakamura H., Shimizu M. (1974). Early and delayed phases of hind paw edema in rats. Jpn. J. Pharmacol..

[66] Andonova V., Peneva P., Georgiev G.S., Toncheva V.T., Apostolova E.G., Peychev Z., Dimitrova S., Katsarova M., Petrova N., Kassarova M. (2017). Ketoprofen-loaded polymer carriers in bigel formulation: An approach to enhancing drug photostability in topical application forms. Int. J. Nanomed..

[67] Kostadinov I., Delev D., Petrova A., Stanimirova I., Draganova K., Kostadinova I., Murdjeva M. (2014). Study on anti-inflammatory and immunomodulatory effects of clomipramine in carrageenan- and lipopolysaccharide-induced rat models of inflammation. Biotechnol. Biotechnol. Equip..

[68] Ohgi Y., Futamura T., Kikuchi T., Hashimoto K. (2013). Effects of antidepressants on alternations in serum cytokines and depressive-like behavior in mice after lipopolysaccharide administration. Pharmacol. Biochem. Behav..

[69] de Carvalho A.M., Rocha N.F., Vasconcelos L.F., Rios E.R., Dias M.L., Silva M.I., de França Fonteles M.M., Filho J.M., Gutierrez S.J., de Sousa F.C. (2013). Evaluation of the anti-inflammatory activity of riparin II (O-methil-N-2-hidroxi-benzoyl tyramine) in animal models. Chem. Biol. Interact..

